# REPRESENTATION OF RURAL OLDER ADULTS IN AI HEALTH RESEARCH: A SYSTEMATIC REVIEW

**DOI:** 10.1093/geroni/igae098.0833

**Published:** 2024-12-31

**Authors:** Kristina Shiroma, Jacqueline Miller

**Affiliations:** Louisiana State University, Baton Rouge, Louisiana, United States; Lousiana State University, Baton Rouge, Louisiana, United States

## Abstract

The demographic shift towards an aging population is particularly pronounced in rural areas, where access to healthcare and social services can be limited. Artificial intelligence (AI) has emerged as a promising avenue for transforming healthcare, particularly for older adults. However, concerns exist regarding equitable access and representation, particularly for geographically isolated, marginalized populations in rural areas. This systematic review investigates the existing research on AI interventions designed to support older adults and their healthcare needs. We searched key health and information science databases for peer-reviewed studies published between 2013 and 2023 that explored AI research with and for older adults. Our systematic analysis identified a robust body of research on AI for older adults. However, a critical gap emerged: a dearth of studies explicitly focusing on older adults in rural communities. While some studies included geographically diverse samples, data disaggregation to examine the specific experiences of rural older adults was challenging to identify. This lack of representation raises concerns about the generalizability of findings and the potential for exacerbating existing healthcare disparities in rural areas. The review underscores the need for research that specifically addresses the unique needs and challenges faced by older adults living outside of urban centers. Future scholarship should prioritize targeted recruitment strategies for rural participants; develop culturally-sensitive AI policies, practices, and products; and explore solutions that address limited representation in these communities. By working to bridge the digital divide through the future of AI research, we can achieve inclusive technology for older adults.

